# Long-term behavioral tracking of *Paramecium bursaria*

**DOI:** 10.17912/micropub.biology.001569

**Published:** 2026-02-07

**Authors:** Azusa Kage, Hiroyuki J. Kanaya

**Affiliations:** 1 Graduate School of Engineering, Muroran Institute of Technology, Muroran, Hokkaido, Japan; 2 Department of Systems Pharmacology, Graduate School of Medicine, The University of Tokyo, Bunkyo-ku, Tokyo, Japan; 3 Institute of Biomedical Engineering, Institute of Science Tokyo, Bunkyo-ku, Tokyo, Japan

## Abstract

The ciliate protozoan *Paramecium* exhibits complex behaviors in response to environmental cues. Here we report a method that enables long-term observation (over 24 hours) of *Paramecium* with a simple experimental procedure. We observed the behavior of *Paramecium bursaria* , a species of *Paramecium* harboring symbiotic green algae, in gas-permeable chambers, where they exhibited light-dependent changes in behavior. We found that, in the 12-hour light-dark (LD) cycles, *P. bursaria* responds to both the dark-to-light and the light-to-dark transitions in different manners. This method provides a way to evaluate the long-term changes in the behaviors of *Paramecium* and other protists.

**Figure 1. Quantitative analysis of the behavior of Paramecium bursaria:**
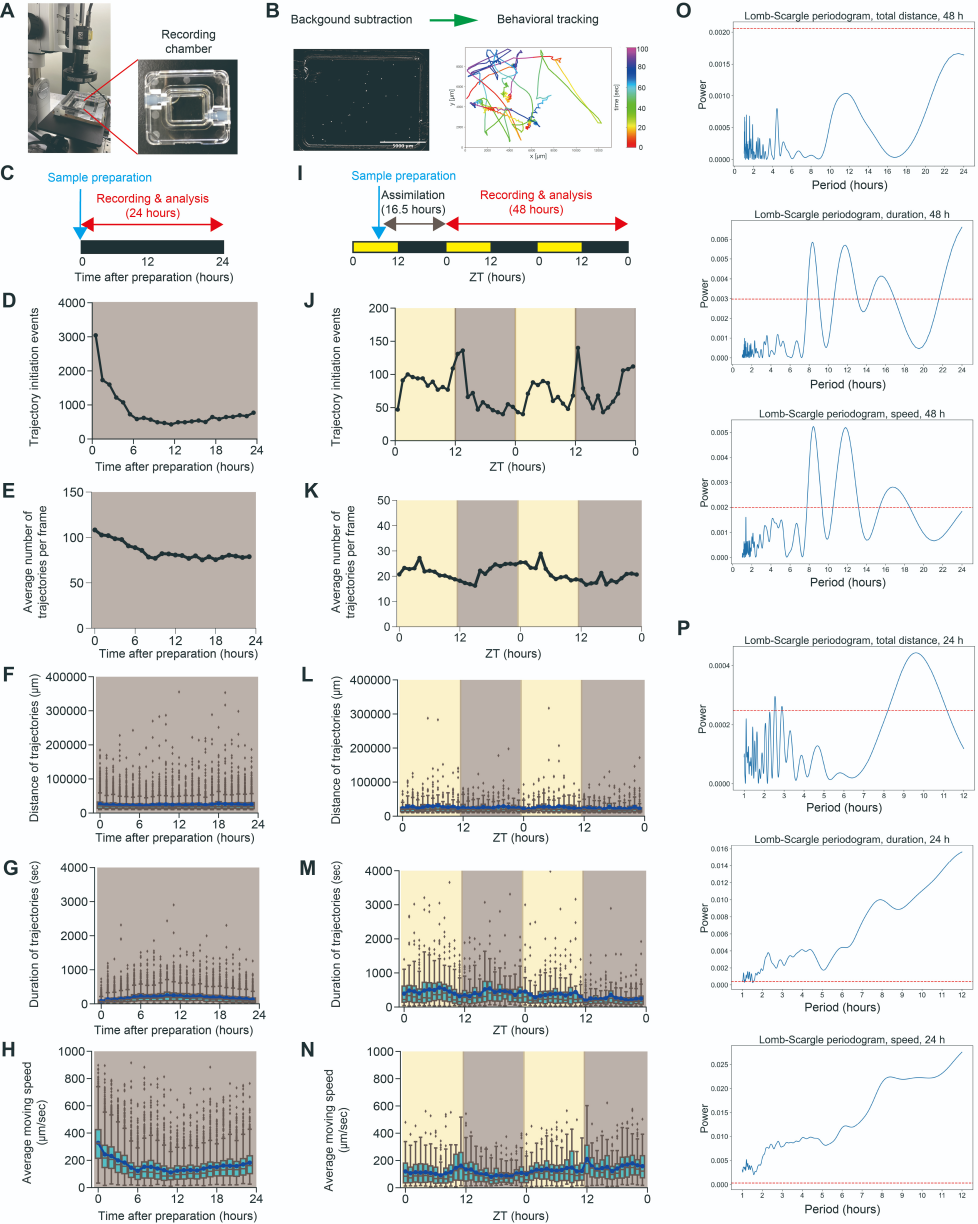
(A) The recording setup. LED lights for the LD cycles were sourced from the enclosure surrounding the chamber. (B) Behavioral tracking. Each trajectory was detected following background subtraction. (C) Experimental scheme of the 24-hour recording. ‘Sample preparation’ indicates the time point when the cells were placed in the chamber. (D) Number of trajectories initiated within 1-hour bins over the 24-hour recording. The data are the sum of triplicate experiments. (E) Hourly average number of active trajectories per frame over the 24-hour recording. The data are derived from the same dataset shown in panel D. (F-H) Total distance (F), duration (G), and the average moving speed (H) of each trajectory. The data are derived from the same dataset shown in panel D. Mean values are plotted as blue points and lines. (I) Experimental scheme of the recording under the 12-hour light-dark (LD) cycle. ZT, zeitgeber time. (J) Number of trajectories initiated within 1-hour bins over the LD cycles. The data are the sum of duplicate experiments. (K) Hourly average number of trajectories per frame over the LD cycles. The data are derived from the same dataset shown in panel J. (L-N) Total distance (L), duration (M), and the average moving speed (N) of each trajectory. The data are derived from the same dataset shown in panel J. Mean values are plotted as blue points and lines. (O, P) The Lomb-Scargle periodograms for total distance, duration, and the average moving speed of trajectories, using 48-hour recording data (O) and 24-hour recording data (P). The red dashed lines represent the 99% lines of the permutation test (n=1000 for each), indicating that peaks above these lines have only a 1% probability of appearing by chance. The periodogram analysis was performed using the Python package scipy. For the box plots, the box ranges from the first to third quartiles; horizontal lines indicate median values; whiskers extend to the minimum or maximum values of 1.5×interquartile range.

## Description

To conduct long-term observation of *P. bursaria* behavior, we used a gas-permeable chamber provided by the Japan Aerospace Exploration Agency and the Japan Space Forum that was originally developed for space experiments (Honda et al., 2025). The chamber is made of polystyrene and enables continuous exchanges of gases, including oxygen ( Fig. 1A ). The chambers were filled with the culture medium containing dozens of *P. bursaria* individuals. The behavior was continuously monitored by capturing images of the chamber at 1-second intervals. Following background subtraction, bright spots ( *P. bursaria* ) were detected using the Python package Trackpy, and the detected spots were linked to form trajectories ( Fig. 1B ). The trajectories with a length of 10 mm or more were used for analyses. As described in previous studies of other *Paramecium* species (Brette, 2021), *P. bursaria* exhibits variable rather than constant behavioral patterns, including straight swimming with directional changes upon encountering obstacles, spiral movements, and even immobile states ( Fig. 1B ).

&nbsp;

To diminish the effect of light, we applied infrared light (peak wavelength: 850 nm) as a background light source, as *P. bursaria* was reported to behaviorally respond to visible light (Iwatuski and Naitoh, 1981; Iwatsuki and Naitoh, 1988). In this basal condition (dark-like condition), we recorded the behavior of *P. bursaria* for 24 hours ( Fig. 1C ). We then quantified the trajectory initiation events per 1-hour bin ( Fig. 1D ), the average number of trajectories per frame ( Fig. 1E ), the distance of trajectories (μm) ( Fig. 1F ), the duration of trajectories (sec) ( Fig. 1G ), and the average moving speed of each trajectory (μm/sec) ( Fig. 1H ). Notably, as tracking was not continuous throughout the recording period and was fragmented, the hourly trajectory initiation events exceeded the actual number of *P. bursaria* cells. Through the analysis, we found that the number of trajectories gradually decreased, subsequently reaching a steady state after approximately 6 hours. This observation suggests that an increasing proportion of cells becomes stopped and assimilates to the environment of the recording chamber ( Fig. 1D- E). While the distance of trajectories appeared to remain constant throughout the recording ( Fig. 1F ), the duration of trajectories changed ( Fig. 1G ), which led to a trend where the average moving speed gradually decreased ( Fig. 1H ).

&nbsp;

Based on this observation, we subsequently conducted longer observations with visible light cues ( Fig. 1I ). *P. bursaria* were cultured in a 12-hour light-dark (LD) cycle prior to applying them to the experiment. Then, *P. bursaria* cells were set into the chamber at zeitgeber time 7.5 (ZT7.5). After sufficient assimilation for 16.5 hours, recording began at ZT0. In the subsequent 48-hour recording period, we found light-dependent changes in the trajectory initiation events ( Fig. 1J ), which exhibited an increase following light onset (ZT0) and around the light-to-dark transition (ZT12). On the other hand, the effects of light conditions on the average number of trajectories per frame were minimal ( Fig. 1K ). While the distance of trajectories showed no clear trend ( Fig. 1L ), the duration of trajectories showed changes in response to LD cycles ( Fig. 1M ). This was reflected in the change of average moving speed, which peaked around the light-to-dark transition (ZT12) ( Fig. 1N ). Thus, under LD cycles, light-on and light-off signals differentially affect the behavior of *P. bursaria* .

&nbsp;

A previous study observed the circadian changes in the behavior of *P. bursaria* by measuring the time-dependent photoaccumulation (Johnson et al., 1989). In addition, by tracking isolated single cells (Hasegawa et al., 1997), the circadian rhythm in the behavior of *Paramecium multimicronucleatum* was evaluated. Compared to these previous approaches, an advantage of our method reported here is conducting seamless recordings, providing a continuous dataset. Although we conducted the bulk observation of dozens of individuals and did not continuously track each particle across the recording period, this method is not largely affected by cell fission and can be conducted with simple procedures without cell isolation. The additional advantage of our method is that we have established a pipeline for automatic image acquisition and analysis. Nakajima and Nakaoka (1989) recorded the swimming behavior of *P. bursaria* under light/dark phases. Although there is no detailed description of the quantification of swimming in this previous report, it is reasonable to assume that they had to put in an enormous amount of effort to quantify swimming, given the technological limitations of the time. Our method requires no human effort to analyze the acquired images.

&nbsp;

In this study, we observed that *P. bursaria* responds to light-on and light-off signals under LD cycles. While the number of trajectories increased around both the light-on (ZT0) and light-off (ZT12) signals, the peak of the average moving speed was observed around the light-off (ZT12). A previous study showed that a decrease in light intensity triggers an avoidance reaction in *P. bursaria* (Saji and Oosawa, 1974), which could contribute to the photoaccumulation of the cells (Cronkite and Van Den Brink, 1981). The observed peak around ZT12 may reflect the photo-seeking property. Interestingly, however, while the peak in the number of trajectories was at ZT12 or ZT13, the increase began at ZT11 with the light still on ( Fig. 1J ). Given that *P. bursaria* possesses an endogenous circadian rhythm with the behavioral output (Johnson et al., 1989), they may exhibit autonomous behavioral changes that are regulated by their endogenous circadian rhythm. In our data, the day-night rhythms of swimming speed were less marked compared to Nakajima and Nakaoka (1989). Nakajima and Nakaoka (1989) reported that swimming almost ceased from the middle of the light phase through the dark phase. However, our analysis still detects movement during the dark phase ( Fig. 1I- N), though we acknowledge differences in both measurement and analysis methods. Regarding the analysis, we conducted background subtraction for tracking, which prevents the tracking of a continuous immobile state. Additionally, for the recording setup, Nakajima and Nakaoka (1989) used glass chambers, while we used the gas-permeable chamber. This difference in chamber materials could potentially affect the observed behavior. Alternatively, light intensity might affect swimming. Further studies should include the effect of light intensity on long-term swimming.

&nbsp;

Given that tracking in our analysis is not continuous and is fragmented, we cannot entirely exclude the possibility that extremely rapid movements may not be annotated as continuous tracks. To assess the effect of fragmentation on our results, we performed the Lomb-Scargle periodogram analysis, which can detect periodicity. The periodogram did not detect a significant peak for the trajectory distance in the 48-hour data, while several significant peaks, including a 12-hour peak, were observed in both the duration of trajectories and the average moving speed ( Figure 1O ). This suggests that under the LD cycles, the observable periodic changes in swimming behavior are primarily reflected in the duration and speed of movement, rather than in the total distance of each trajectory. In the 24-hour data, on the other hand, we detected significant peaks in total distance, duration, and speed ( Figure 1P ). These might be artifacts or endogenous rhythms that are masked under the LD cycle. Although further studies are required to elucidate this point, we state that the effect of fragmentation is limited at least under the LD cycle.

&nbsp;

Additionally, based on our current data, we cannot rule out the possibility that the heating effects of the recording system and the remaining sensitivity of *P. bursaria* to infrared light affect our results. In our method, however, the infrared light signal was continuously provided as the background, and the visible light was given additively during the light phases. Thus, the observed behavioral patterns are considered to be brought about by the visible light source. While this report is limited to the analysis of the influence of LD cycles, the method will also be applicable to the analysis of a variety of behaviors of *Paramecium* and other protists that occur on a long-time scale.

## Methods

**Strain**

*Paramecium bursaria* strain HA1g was obtained from NBRP Paramecium Laboratory, Yamaguchi University, Japan. The cells were co-cultured with the bacteria *Klebsiella aerogenes* (ATCC 35028) in the medium containing lettuce juice (500 g of raw lettuce per 1 L) and modified Dryl’s solution (2 mM Na _2_ C _6_ H _5_ O _7_ , 1.4 mM Na _2_ HPO _4_ , 0.6 mM KH _2_ PO _4_ ) (Mikami, 1980) at a ratio of 1:40 at 20°C under a 12-hour light-dark cycle. Cells after 3-13 days of inoculation were used for long-term tracking.

&nbsp;

**Behavioral recording**

The chambers used for long-term observation were kindly provided by the Japan Aerospace Exploration Agency and the Japan Space Forum (dimensions: 14 mm × 10 mm × ~1 mm) (Honda et al., 2025). The recording chamber was filled with the culture medium containing *P. bursaria* individuals (approximately 40 for the 24-hour recordings and approximately a dozen for the LD recordings), using syringes equipped with a 23-gauge needle (Terumo, Tokyo, Japan). The recording was performed in an isolated space where ambient room light could not interfere. For dark-field imaging, an infrared camera (STC-MBS1242U3V, Omron Sentech, Ebina, Japan) with a fixed-focus lens (VS-TCT05-65/S, VS Technology, Tokyo, Japan) and a ring LED light with a peak wavelength of 850 nm (UDR-50IR90-850, U-Technology, Tokyo, Japan) was used. Images were captured every 1 second for 24 to 48 hours and stored as JPEG files (compression rate: 75). The total file size for 24 hours recording data was approximately 40 GB. For the experiments in the LD cycle, a LED light source (Lepro, Innovation Inc., Las Vegas, USA; color temperature: 6000 K) was provided from outside the imaging area.

***&nbsp;***

**Tracking analysis**

For the detection of *P. bursaria* individuals, the Python package Trackpy (https://soft-matter.github.io/trackpy/) was used. JPEG images were read one by one with OpenCV, and the averages of 100 images were used for background subtraction to diminish the effect of non-moving objects like debris and the edge of the chamber. The bright points were detected from the subtracted images by the *locate* function of Trackpy. The data of bright points were kept in memory as pandas DataFrame and then linked to form trajectories with the *link* function of Trackpy. The analysis was performed using Alienware m15 R7 (Dell, Round Rock, TX, USA) with 64 GB of RAM. To reduce computation time, we reduced the size of the original images (4000×3000 pixels) by a factor of 0.3 when necessary. The trajectories with a length of 10 mm or more were used for the analysis. Then, the number of trajectories and the average moving speeds were calculated. For the number of trajectories, the time points to initiate each trajectory were identified and counted for each 1-hour bin. The average moving speeds were calculated as the mean speed for each trajectory within each 1-hour bin.

&nbsp;

**Code and data availability**

The code for this study is available at https://github.com/kageazusa/paramecium-long-term-tracking. The original dataset of the 24-hour recording ( Fig. 1C- H) is available at https://zenodo.org/records/16743472.
